# Anemia among children aged 2–5 years in the Gaza Strip- Palestinian: a cross sectional study

**DOI:** 10.1186/s12889-015-1652-2

**Published:** 2015-04-01

**Authors:** Rima Rafiq El Kishawi, Kah Leng Soo, Yehia Awad Abed, Wan Abdul Manan Wan Muda

**Affiliations:** School of Public Health, Al Quds University, Gaza City, Gaza Strip Palestine; Department of Nutrition, School of Health Sciences, Health Campus, Universiti Sains Malaysia, Kubang Kerian, Kelantan 16150 Malaysia

**Keywords:** Anemia, Hemoglobin, Prevalence, Associated factors

## Abstract

**Background:**

Anemia is a major public health problem worldwide, with adverse consequences on child growth, development, and survival. This deficiency has affected approximately a quarter of the world population. This study aimed to determine the prevalence of anemia and the associated factors among preschool children in the Gaza Strip.

**Methods:**

A cross-sectional study was conducted between May and September 2012. A total of 357 preschool children were selected using multistage sampling method from Jabalya refugee camp, El Remal urban area, and Al Qarara rural area. Hemoglobin level was measured, and anemia diagnosis was confirmed at a level <11.0 g/dL. In this study, we utilized a pretested questionnaire for face to face interview with mothers. Anthropometric indicators for children were measured using the WHO guideline. Descriptive and multivariate analyses were conducted to determine the prevalence and associated factors of anemia.

**Results:**

The overall prevalence of anemia was 59.7% among preschool children in the Gaza Strip, 46.5% and 13.5% of which are mild and moderate, respectively. The mean hemoglobin level was 10.83 ± 0.86 g/dl. Children living in Jabalya refugee camp have a high risk of anemia [adjusted b= −0.55; 95% confidence interval (CI;-0.72,-0.39); *p < 0.001*]. Boys were more susceptible to this deficiency than girls [adjusted b = 0.17; 95% CI (0.0.01, 0.33); *p = 0.031*]. Hemoglobin level increased with age [adjusted b = 0.02; 95% CI (0.01, 0.03); *p <* 0.001]. Hemoglobin level decreased in children living in poor households [adjusted b = −0.24; 95%CI (−0.41,-0.06); *p = 0.006*]*.* Underweight children were more susceptible to anemia than normal weight children [adjusted b = − 0.22; 95% CI (−0.41, −0.03); *p = 0.025*]*.*

**Conclusions:**

The prevalence of anemia among preschool children in the Gaza Strip was higher than those reported in previous local studies, indicating that anemia is a major public health problem. In this study, we also observed mild and moderate cases among children, whereas severe anemia was not observed. Independent predictors of anemia were geographic location, sex, age, monthly income, and malnutrition. Results provided the baseline information on anemia, therefore, especial attention should be given on intervention of anemia.

## Background

Anemia is a clinical condition associated with multiple causes, the most common of which is nutritional causes, such as iron, folate, vitamin B12, and protein deficiencies. Non-nutritional causes, such as congenital factors and parasitic diseases, also exist [1]. According to the World Health Organization database on anemia for 1993–2005, the global estimated prevalence of anemia was 25.0%, in which 47.4% of preschool children were anemic [2,3]. Children and women of reproductive age have the highest risks of anemia because of their physiological vulnerability [3]. Nutritional deficiency anemia is the most prevalent deficiency in the world, with approximately one billion cases of iron deficiency anemia [4]. In children, iron deficiency is primarily due to increased iron requirement as they grow; meanwhile, the major causes of iron deficiency among women of reproductive age are menstrual blood loss and pregnancy [5]. Anemia has adverse health consequences in preschool children, which include effects on cognitive development, school performance, physical growth, and immunity [6,7]. The distribution of anemia in a population is inversely associated with economic development [8]. The economy of the Gaza Strip is mainly dependent on external aid and on the tunnel with Egypt after the war from 2008 to 2009. Socioeconomic deterioration is associated with the increasing poverty level among Palestinians in the Gaza Strip, which resulted from the siege and the external isolation by the Israeli government [9]. Such deteriorated conditions have negatively affected the population, particularly children. A cross-sectional baseline survey was conducted to obtain data and develop a policy-based approach to control anemia among children in the Gaza Strip. This study aimed to determine the prevalence of anemia among children aged two years to five years in three different geographic areas in the Gaza Strip and detect the associated factors of this deficiency.

## Methods

This study was conducted between May 2012 and September 2012 in the Gaza Strip to determine the prevalence and associated factors of anemia in children aged 2–5 years using questionnaires, anthropometric indices, and blood tests.

### Study location

**Figure 1:**
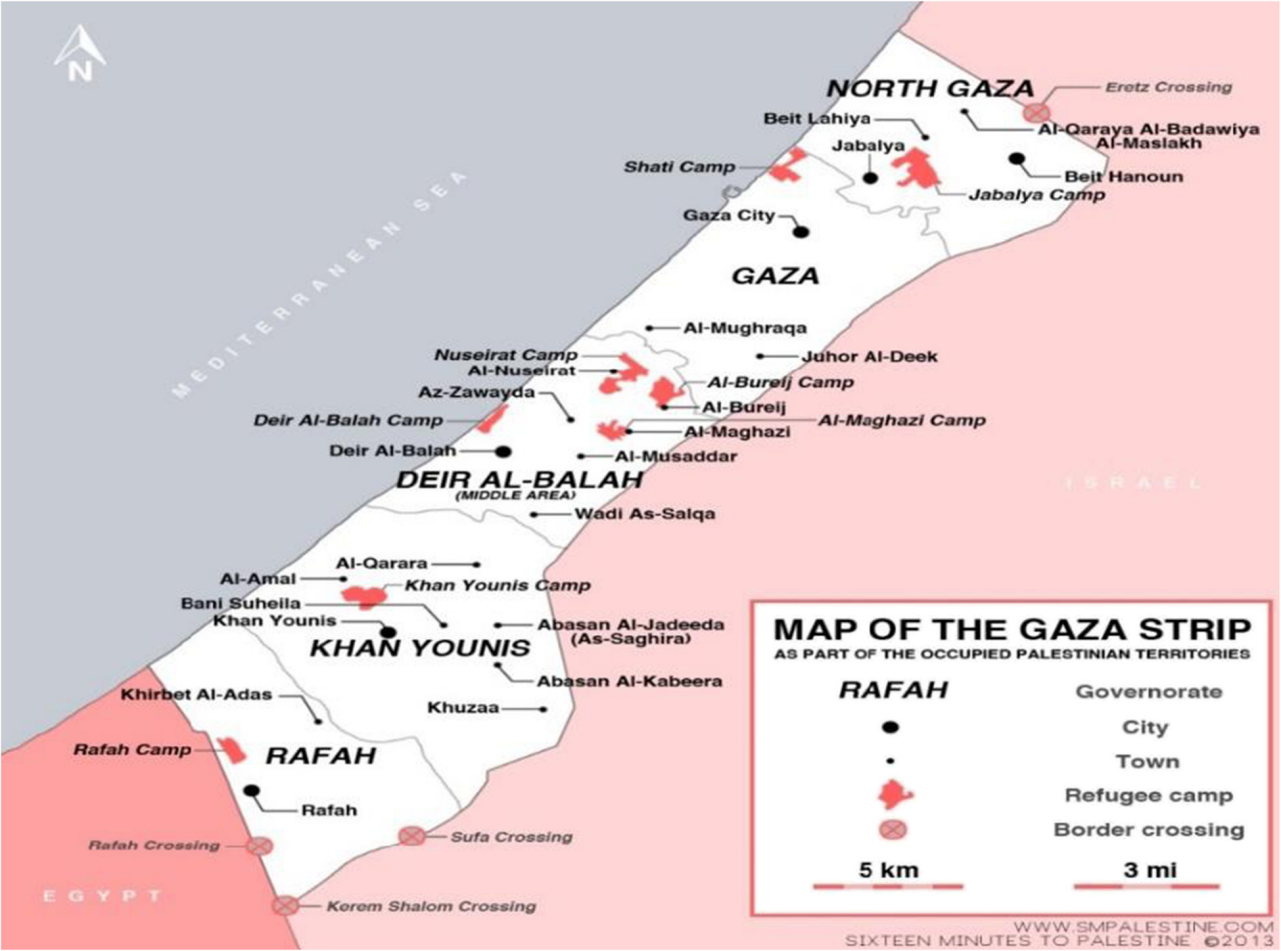
**Map of the Gaza Strip.**

This study was conducted in Jabalya refugee camp in the north of Gaza Strip, El Remal urban area in Gaza city, and Al Qarara rural area in the south of Gaza Strip (Figure 1).

### Sample size

The single proportion formula in the Epi-Info Software Revision (Version 2; 2002) was used to determine the required sample size and it came out to be 334. Accounting for attrition rate 20.0%, a total of 400 participants had to be recruited for the study. The researcher visited 357 out of 400 households in the three areas in the Gaza Strip, to obtain a response rate of 89.2%.

### Sampling method

Out of the total population in the three areas, preschool children comprised of 19.1% of the respondents. The number of households chosen for each area was weighted in proportion to the total population of preschool children as shown in Table 1:

**Table 1:** **Sample distribution**

**Area**	**Estimated population**	**Estimated preschool children**	**Sample**
Jabalya (Refugee Camp)	110,000	21,010	226
El Remal (Urban area)	70,000	12,370	133
Al Qarara (Rural area)	20,000	3,820	41
Total	200,000	37,200	400

The researcher selected three areas depending on sociodemograhic situation, namely, Jabalya refugee camp in the north of Gaza Strip, El Remal in Gaza city, and Al Qarara in the south of Gaza Strip. At the first stage, a random sample of clusters was chosen from the El Remal (urban area) and Jabalya camp (refugee camp) representing areas of high population density, then from the Al Qarara (rural area), representing areas of lesser population density. At the second stage, systematic households were selected within each cluster in urban, camp, and rural areas. In these households with more than one child aged 2–5 years, the youngest child was selected. Children who met the inclusion and exclusion criteria were identified; they did not suffer from psychomotor retardation, hormonal disorders, chronic debilitating diseases, congenital heart diseases, or acute severe illnesses. A total of 217, 100 and 40 children from Jabalya refugee camp, El Remal urban area, and Al Qarara rural area, respectively, were selected.

### Data collection

Structured questionnaire was used in this study, mothers were interviewed about socio-demographic information, child feeding practice such as breastfeeding, exclusive breastfeeding, drinking tea, in addition supplements such as iron syrup and vitamins. The validity of the questionnaires was also tested to seven experts in the field of nutrition and health, and worked on all comments and advices. Prior to conducting the study, pilot testing among 30 mothers was conducted and the questionnaire was adjusted to check and to evaluate the response of participants.

### Anthropometric indicators

#### Weight

Weight of the child was measured with a SECA digital weighing scale to 0.1 kg precision. The researcher adjusted the scale before each measuring session, and accuracy was checked by comparing the scale reading with a known weight. Child was weighed barefooted, wearing only underwear. The measurements were taken twice and the average was calculated.

#### Height

The height was measured using a SECA body meter with 0.1 cm precision. Height measurements were taken without shoes; the children stood against a wall with feet flat on their base, heels, buttocks, shoulders, and their backs of the head touching the wall and head positioned looking straight ahead. The mean of two measurements was calculated. Birth date was recorded from the birth certificates.

Raw anthropometric data were transformed into Z-scores using the program WHO ANTHRO (2.2, January 2011). To assess children’s nutritional statuses, Z-scores were calculated according to the WHO child growth standards for children. Weight for age was used to denote underweight, WAZ of Z < −1.0. Height for age was used to denote stunting, HAZ of Z < −1.0, and weight for height was used to denote wasting WHZ of Z < −1.0 [10].

#### Blood test

##### Blood

Samples were collected by a laboratory technician, and were obtained through the vein after cleansing the antecubital area with 70.0% alcohol and medical cotton using a butterfly needle. The blood was drawn using anticoagulant tube contained ethylene diamine tetraacetate (EDETA), the date, and the serial numbers were recorded on each tube. Measurement of hemoglobin level was performed using electronic equipment HORIBA ABX Micros ES60 after mixing the samples. The three main physical technologies used in hematology analyzers are: electrical impedance, flow cytometry, and fluorescent flow cytometry. These are used in combination with chemical reagents that lyse blood cells to extend the measurable parameters. The widely accepted combination of mild, moderate, and severe anemia categories was commonly used, that ranged from (10.0 g/dl to 10.9 g/dl), (7.0 g/dl to 9.9 g**/**dl), and less than 7.0 g/dl, respectively [11].

### Statistical analysis

Data were analysed using Statistical Package for the Social Sciences (IBM SPSS version 20.0). We used frequency and proportion, as well as means and standard deviation to describe the characteristics of the study sample, and to estimate the prevalence of anemia in the Gaza Strip, and in the three locations*.* Linear regression was performed to control for confounding factors. The initial step of the multivariate analyses was Single linear regression (SLnR) to examine the relationship between each of the independent variables and the dependent variable (Hemoglobin level), and include the results of independent variables with *p* < 0.25 (i.e., geographic location, sex, employed mother, mother’s educational level, monthly income, household assistance, and drinking tea,). Subsequently a Stepwise multiple linear regression (MLR) procedure was used to progressively exclude independent variables, retaining only those with a significance level of p < 0.05.

### Ethical issues

Ethical approval to conduct the study was obtained from the Helsinki Committee in Ministry of Health in the Gaza Strip, and from Universiti Sains Malaysia (USM) ethical Committee, in which the purpose of the study was clarified. Written consent form was obtained from each mother before interview, and mothers were informed about voluntarily answering of the questionnaires and the assurance of anonymous and confidential information. Written permissions of the mothers were first required for blood collection from their children, including that child’s sample would be used anonymously. Moreover, any research results that are published would not identify her child in any way. To increase confidentiality, child’s sample will not have his/her name on it, or information that could reveal child’s identity.

## Results

A total of 357 out of 400 households in these areas were visited, and yielded a response rate of 89.2%. Of the 43 non-respondents, 10 mothers refused to participate in this study, 22 children refused to anthropometric measurements and the blood-drawing procedure, and 11 households excluded children ages 2–5 years. All 357 children voluntarily accepted to participate and give blood sample for analysis.

Table 2 presents the demographic and other social characteristics of the households in the study sample. Participants of this study constituted of 60.8%, 28.0%, and 11.2% of which were from Jabalya refugee camp, El Remal urban area, and Al Qarara rural area, respectively. In terms of socioeconomic status, monthly income was categorized into two categories, one above the poverty line >1400 New Israeli shekel (NIS), and one below the poverty line ≤1400 NIS. Poverty line defined based on family income of US$ 2 or 7.8 New Israeli Shekels (NIS) per person per day in Palestinian territories (exchange rate of 3.9 NIS per US Dollar) [12]. The poverty line was ≤1400 NIS for a family with six persons per household (as average household size was approximately 6.0). Our results showed that 68.8% of the households lived below the poverty line, and 51.8% of the households received food or monetary assistance. The average household size was 6.50 ± 1.99 persons per household. Approximately, more than half of all fathers had medium level of education (preparatory and secondary), while 37.3% had high level of education (graduated and post graduated), while 8.1% continued for low level of education (elementary). Almost 77.0% of fathers had jobs, and 23.0% weren’t working. In terms of education, 72.3% of women in the Gaza Strip obtained moderate educational level (preparatory and secondary), 22.1% received high educational level (graduated and post graduated), and 5.6% illiterate or obtained low educational level (elementary). Majority 95.0% of mothers were housewives, whereas 5.0% were employed.

**Table 2:** **Household characteristics**

**Variables**	**n = 357 (%)**	**Mean (SD)**
**Geographical location**		
El Remal (Urban area)	100 (28.0)	
Al Qarara (Rural area)	40 (11.2)	
Jabalya (Refugee Camp)	217 (60.8)	
**Monthly income (Shekel)**		
Above poverty line >1400	112 (31.4)	
Under poverty line ≤1400	245 (68.6)	
**Household assistance**		
Yes	185 (51.8)	
No	172 (48.2)	
**Household size**		6.5 (1.99)
**Father’s education level**		
High	133 (37.3)	
Medium	195 (54.6)	
Low	29 (8.1)	
**Father’s working**		
Yes	275 (77.0)	
No	82 (23.0)	
**Mother’s education level**		
High	79 (22.1)	
Medium	258 (72.3)	
Low	20 (5.6)	
**Mother’s job**		
Employed mother	18 (5.0)	
Housewife	339 (95.0)	

US$ = 3.90 Shekel.

Table 3 summarizes the characteristics of children, 52.7% and 47.3% of which were male and female. The mean age of children in the sample was 39.58 ± 10.74 months. Child birth order was categorized as 1–4 and >4**;** more than half of the children 61.6% were between the first sibling and the fourth, whereas 38.4% of children were after the fourth sibling. Majority of the children 97.8% were breastfed, whereas 24.4% received breastfeeding for six months as per WHO exclusive breastfeeding recommendation. Results showed that higher early weaning practice in case of female children compared to male children. More than half of the children 52.9% had tea with meals, and majority of the children (83.5% and 85.4%) did neither receive iron syrup nor vitamins, respectively, during the last six months of the interview that may be possibly related to availability of the drug, and affordability.

**Table 3:** **Children’s general characteristics**

**Variables**	**n = 357 (%)**	**Mean (SD)**
**Sex**		
Male	186 (52.7)	
Female	169 (47.3)	
**Age (month)**		39.58 (10.74)
**Child Birth Order**		
1-4	220 (61.6)	
>4	137 (38.4)	
**Breastfeeding**		
Yes	349 (97.8)	
No	8 (2.2)	
Exclusive Breastfeeding*		
Yes	87 (24.4)	
No	270 (75.6)	
**Weaning (month)**		
Male		15.2 (6.4)
Female		14.3 (6.4)
**Drinking Tea**		
No	168 (47.1)	
Yes	189 (52.9)	
**Iron Syrup**		
Yes	59 (16.5)	
No	298 (83.5)	
**Vitamins A & D**		
Yes	52 (14.6)	
No	305 (85.4)	
**Weight (kg)**		14.20 (2.42)
**Height (cm)**		94.14 (7.94)
**Hemoglobin (g/dl)**		10.83 (0.86)

*Exclusive breastfeeding: up to six month.

Hemoglobin levels and anthropometric measurements were used to measure nutritional status of the children in the three areas in the Gaza Strip. The mean hemoglobin level, the mean weight, and the mean height among the children aged 2–5 years were 10.83 ± 0.86 g/dl, 14.20 ± 2.42 kg, and 94.14 ± 7.94 cm. Table 4 shows the prevalence of anemia among children was 59.7%, approximately 46.5% and 13.2% of whom suffered from mild and moderate anemia. Severe anemia was not recognized among children. Majority of children 74.8% were categorized under normal weight (Z-score-1.0 to 2.0), while 24.4%were underweight (Z-score < −1.0), the lowest proportion for overweight children (Z-score > 2.0) was 0.8%. Among the children, approximately half of them were stunted 52.4% (Z-score < −1.0), and 47.6% were normal, while the wasted comprised 8.4% (Z-score < −1.0) and the majority were normal 87.0%. Table 5 shows the highest prevalence rates of anemia 70.0% was among refugees’ children in Jabalya refugee camp, followed by 52.5%, in Al Qarara rural area, while the lowest 40.0% was in El Remal urban area. Table 6 presents the associated factors with the hemoglobin level, in which the prevalence of anemia in the Gaza Strip among preschool children was associated with location, age, sex, family income, and malnutrition. Children in Jabalya Refugee camp have high risk of anemia [adjusted b = −0.55; 95% CI (−0.72,-0.39); *p < 0.001*]. Significant sex difference with anemia existed, with female children having higher hemoglobin level compared with male children [adjusted b = 0.17; 95% CI (0.01, 0.33*)*; *p = 0.031*]**.** The age and the hemoglobin level obeyed a significant linear positive relationship. Those who were older have a higher hemoglobin level of 0.02 g/dl [adjusted b = 0.02; 95% CI (0.01, 0.03); *p < 0.001*]. Hemoglobin level decreased in children who lived in low income households [adjusted b = −0.24; 95% CI (−0.41,-0.06); *p* = 0.006]. Anemic children were significantly associated with being underweight [adjusted b = −0.22; 95% CI (−0.41, −0.03); *p = 0.025*]. Birth order, maternal employment and educational level, father employment, household assistance, breastfeeding status, tea drinking, intake vitamin supplements, stunting, and wasting were not associated with hemoglobin level (*p > 0.05*; results not shown).

**Table 4:** **Nutrition status among children under-five years in the Gaza Strip**

**Variables**	**n = 357 (%)**
Hemoglobin concentration*	
Normal Hemoglobin (g/dl) ≥11.0 g/dl	144 (40.3)
Mild anemia (10.0-10.9) g/dl	166 (46.5)
Moderate anemia (7.0-9.9) g/dl	47 (13.2)
Weight for age**	
Normal-1.0-to 2.0	267 (74.8)
Underweight < −1.0	74 (24.4)
Overweight > 2.0	3 (0.8)
Height for age**	
Normal-1.0-to 2.0	170 (47.6)
Stunting < −1.0	187 (52.4)
Weight for height**	
Normal-1.0-to ≤2.0	311 (87.1)
Wasting < −1.0	30 (8.4)
High > 2.0	16 (4.5)

* WHO [11].

** WHO [10].

**Table 5:** **Prevalence of anemic children in the three areas in the Gaza Strip**

**Variables**	**Urban**	**Rural**	**refugee camp**
	**n = 100 (%)**	**n = 40 (%)**	**n = 217 (%)**
**Anemia** Hb < 11.0 g/dl	40 (40.0)	21 (52.5)	152 (70.0)
**Normal Hemoglobin**	60 (60.0)	19 (47.5)	65 (30.0)

**Table 6:** **Associated factors of child’s HB level in the Gaza Strip**

**Variables**	**Simple Linear Regression**		**Multiple linear Regression**	
	**b** ^**a**^ **(95%CI)**	**P-value**	**b** ^**b**^ **(95%CI)**	**P-value**
Geographic location_0_	−0.54 (−0.71,-0.36)	<0.001	−0.55 (−0.72,-0.39)	<0.001
Geographic location _1_	0.54 (0.35,0.73)	<0.001	-	-
Sex	0.16 (−0.11,0.34)	0.066	0.17 (0.01,0.33)	0.031
Age (months)	0.02 (0.01,0.02)	<0.001	0.02 (0.01,0.03)	<0.001
Birth order	−0.001 (−0.04,0.03)	0.932	-	-
Employed mother	0.27 (−0.13,0.68)	0.181	-	-
Father working	−0.04 (−0.25,0.17)	0.708	-	-
Mother’s education_0_	0.26 (0.05, 0.48)	0.015	-	-
Mother’s education_1_	−0.08 (−0.47,0.31)	0.684	-	-
Income under poverty line (≤1400 Shekel)	−0.26 (−0.45,-0.07)	0.008	−0.24 (−0.41,-0.06)	0.006
Household Assistance	0.33 (0.15,0.50)	<0.001	-	-
Exclusive Breastfeeding	0.11 (−0.10,0.31)	0.312	-	-
Drinking tea	−0.17 (−0.35,0.005)	0.056	-	-
Vitamins supplements	0.33 (0.08, 0.58)	0.009	-	-
Underweight	−0.19 (−0.40,0.01)	0.061	−0.22 (−0.41,-0.03)	0.025
Stunting	−0.27 (−0.44,0.01)	0.003	-	-
Wasting	−0.07 (−0.39,0.25)	0.67	-	-

^a^ Crude regression coefficient.

^b^ Adjusted regression coefficient.

Stepwise multiple linear regression method was applied. R2 = 20.6%.

Geographic location: Urban area is the reference.

Geographic location0: Jabalya refugee camp.

Geographic location1: Rural area.

Sex: Male is the reference.

Educational High level is the reference.

Income above poverty line (>1400 Shekel) is the reference group, US $ = 3.90 Shekel.

Household with assistance is the reference group.

## Discussion

Poverty and the strict blockade of goods (including those for export and the flow of products, services, and people) have increased food insecurity and have remained virtually unchanged [13]. The prevalence of anemia among preschool children in the Gaza Strip has reached 59.7%, and being ≥40.0, is considered severe according to anemia classification and should be recognized as a major problem of public health [2]. Malnutrition and anemia among children aged two years to five years are still major public health problems that contribute to child morbidity and mortality, particularly in the Jabalya refugee camp, and should thus be addressed with an effective intervention. The highest prevalence of anemia was observed in the Jabalya refugee camp, followed by Al Qarara rural area and El Remal urban area in Gaza city. These observations are attributed to crowded living conditions and poverty, in which the chronic imbalance between the needs of the refugee population and food assistance increased the prevalence of malnutrition. Results from the Nutritional Assessment of the West Bank and Gaza Strip in 2003 showed that 71.8% of the population in the Gaza Strip, especially in the Jabalya refugee camp, received food assistance from humanitarian agencies (e.g., United Nations Relief and Works Agency and World Food Program) [14]. This assistance has temporarily alleviated malnutrition, although the economic situation has virtually collapsed as a result of increasing unemployment and poverty, which have long-term adverse effects on the nutritional status of young children. In addition rural community had high proportion of food-insecure households and the constant clashes of this area with Israel along the border has led to the moderate to poor socioeconomic conditions in this community as well as the high prevalence of malnutrition [15]. Our results revealed that the prevalence of anemia among children aged two years to five years in the Gaza Strip was higher compared with local previous studies [15,16]. This result indicated that the prevalence of anemia has not decreased and should thus be considered a major public health problem in Palestinian territories. Multivariate analysis revealed that the sociodemographic characteristics (such as geographic location, sex, age, and monthly income) and nutritional status of children were significantly associated with the prevalence of anemia. Children living in the Jabalya refugee camp have a high risk of anemia, which is attributed to poverty and unemployment that have negative effects on the nutritional status of preschool children, especially refugee children [14,17]. Our results revealed that male children were more susceptible to anemia than female children. Similar findings have been documented in a previous study, which revealed that the prevalence of anemia increased among male children [18]. These differences can be attributed to genetics; otherwise, an increased incidence of iron deficiency in boys [19]. Previous studies reported a high prevalence of iron deficiency anemia among infants who continued late weaning [20]. Sex differences in feeding practices were observed in this study. Our results indicated that male children were breastfed for a longer period than female children. Girls consumed more complementary foods than breast milk. Food diversity was protective of anemia [19]. Late introduction of weaning foods has been associated with iron deficiency anemia in infants one year to two years of age [21]. Among the factors analyzed, age had an important influence on anemia. The results of this study showed that hemoglobin concentration increased with age. Among preschool children in Haiti, an association between hemoglobin and age was observed and showed that children were able to eat a more varied diet with an increase in age [22]. Poverty has evidently contributed to food and nutrition insecurity [4]. Our data showed that income (from salaries or social programs) was significantly associated with hemoglobin level. Based on our results, low hemoglobin concentration was observed in children who live in poor households, which is consistent with previous information that children living in low-income (≤1400 NIS) households have a higher risk of anemia [23,24] because of inadequate diet, limited access to basic needs (e.g., health services), and high susceptibility to infectious diseases [4 16]. The emergence of malnutrition was also an important public health concern in the study area. Examination of growth abnormalities resulting from malnutrition revealed that the hemoglobin concentration decreased among underweight children. Another study showed that stunted children were more susceptible to anemia [15,25].

One limitation of this study is the cross-sectional design that did not determine exactly the different effects of risk factors on anemia patterns among children in the Gaza Strip. Case–control studies should be modified and developed in the future to address the risk factors. Moreover, additional studies on micronutrient deficiency anemia, such as iron, vitamin B12, and folic acid deficiencies, are needed.

## Conclusion

The findings of this study showed the widespread occurrence of anemia among preschool children in the Gaza Strip, with prevalence rates substantially higher compared with previous local studies. The high prevalence of anemia can be attributed to sociodemographic factors and child malnutrition. The results of this study indicated geographic location differences in the prevalence of anemia in children, with the highest rate observed in the Jabalya refugee camp. Young children were more likely to develop anemia than older children. Boys were more susceptible to anemia than girls. Households with low income were more likely to have anemic children. In addition, underweight children were observed to be more anemic than normal weight children. Therefore, improving the economic status of the society and alleviating poverty are essential strategies to reduce the prevalence of anemia. In addition, the weaning practices of the mothers were inappropriate. Antenatal clinics should educate mothers on appropriate child feeding practices.
